# Association analysis of variant near *ZNF389* with ewe cumulative production in three sheep breeds

**DOI:** 10.1111/age.12161

**Published:** 2014-04-11

**Authors:** S N White, M R Mousel, M V Gonzalez, M A Highland, L M Herrmann-Hoesing, J B Taylor, D P Knowles

**Affiliations:** * Animal Disease Research Unit, USDA-ARSPullman, WA, 99164, USA; † Department of Veterinary Microbiology and Pathology, Washington State UniversityPullman, WA, 99164, USA; ‡ U.S. Sheep Experiment StationDubois, ID, 83423, USA

## Description

Genome-wide association identified a gene region including *ZNF389* as highly associated with small ruminant lentivirus (SRLV) proviral concentration among infected sheep.^1^ Within this region, a deletion variant near *ZNF389* was associated with control of SRLV proviral concentration in multiple sheep flocks.^2^ Because proviral concentration is a measure of viral replication also correlated with disease,^3^ this deletion variant may be predictive for SRLV disease severity. However, an open question is whether this deletion variant could also be associated with production or other traits that might enhance or detract from its value in selective sheep breeding.^4^

## Animals and phenotypes

We sampled 764 Idaho Rambouillet, Polypay and Columbia ewes as described.^2^ SRLV testing was performed by validated assay as described^5^ (Appendix S1) to establish lifetime and partial-lifetime SRLV-negative status. Phenotypes included birth weight; weaning weight; mature weights at 3 and 4 years; milk score; udder scores at 3 and 4 years; and lifetime measures of fleece weight, number of lambs born, number born alive, number born dead, weight of lambs born and weight of lambs weaned (Appendix S1).

## Genotypes and analysis

The deletion variant was g.29500068_29500069delAT relative to NC_019477.1 (rs397514112). Genotyping was performed as described^2^ (Table S1). Analysis employed the mixed model or glimmix procedure in sas v9.2 (SAS Institute) as described.^6^,^7^ For each analysis, the production trait of interest was included as the dependent variable. Other model terms included breed, sire, year of birth, age in years, age at last lambing and genotype as described (Appendix S1). Bonferroni correction accounted for multiple testing.

## Comments

The *ZNF389* deletion variant was not consistently associated with any tested production traits between lifetime and partial-lifetime SRLV-negative groups (Tables S1 and S4). Insertion homozygotes previously associated with reduced SRLV proviral concentration were associated with lower birth weight in the partial-lifetime group (Bonferroni *P* = 0.033; Table S4), but the weight difference was small (0.41 kg) and not replicated in the lifetime group (nominal *P* = 0.41; Table S2). Further, there was no association with weaning or later weights (Tables S2 and S4). These results showed no consistent association of the *ZNF389* deletion variant with ewe lifetime production. Other breeds and additional traits, such as wool diameter and infectious disease traits besides control of SRLV, should be examined to more fully assess this locus.

## Supporting information

**Appendix S1** Additional Methods Description. **Table S1** *ZNF389* deletion variant genotype counts among lifetime SRLV-negative ewes. **Table S2** Association results between *ZNF389* deletion variant and production phenotypes in lifetime SRLV-negative ewes. **Table S3** *ZNF389* deletion variant genotype counts among partial-lifetime group SRLV-negative ewes. **Table S4** Association results between ZNF389 deletion variant and production phenotypes among partial-lifetime group SRLV-negative ewes.
