# Supplementary material for: Association analysis of variant near ZNF389 with ewe cumulative production in three sheep breeds
Source: Anim Genet. 2014 Apr 11;45(4):613–4. doi: 10.1111/age.12161 (PMC4171751; doi:10.1111/age.12161)
Supplement: Supplementary file 1 [file age0045-0613-SD1.pdf]

Appendix S1: Additional Methods description.

SRLV testing procedures:

Repeated SRLV testing was performed using a validated qPCR (quantitative Polymerase Chain Reaction) method [Main Text Reference 5]. Additional ewes positive for SRLV on first testing were not included in analysis because there was no time point at which they were known to be SRLV-negative. Ewes identified as SRLV-negative for their entire productive life after approximately annual screening were analyzed in the "lifetime" group. Ewes SRLV-negative at initial testing but later found to be SRLV-positive were analyzed separately in the "partial-lifetime" SRLV-negative group for cumulative production trait association.

5 Herrmann-Hoesing, L.M. *et al.* (2007) *Clin Vaccine Immunol* **14**, 1274-8.

Statistical analysis:

For continuous traits, the mixed model procedure of SAS v9.2 was used with the production trait of interest as the dependent variable. Independent fixed effects included breed, year of birth, and deletion variant genotype. Sire nested within breed was included as a random term. Age in years was included as a linear covariate when not fixed by phenotype definition. Analysis of lamb traits also included age at last lambing as an additional covariate. Udder scores were treated as categorical dependent variables in mixed poisson regression models using the glimmix procedure of SAS v9.2 with independent variables as described above. Bonferroni correction within each group accounted for multiple testing.

Phenotype characterization procedures:

Birth weights, weaning weights, and mature weights at 3 and 4 years of age were actual recorded weights (unadjusted) in kilograms.

Milk score was a subjective assessment by farm staff on the amount of milk available to lambs 12 to 24 hours after birth according to the following scale:

0) None
1) Poor
2) Fair
3) Average
4) Good
5) Very Good

Milk scores were collected each year the ewe lambed, and the milk scores analyzed were from the final year for which data were collected on the ewe.

Udder scores were taken by farm staff each year according to the following scale:

1) Normal but dry (not nursing)
2) Normal with sizable milk cistern
3) Pendulous with large milk cistern
4) Asymmetrical milk cistern
5) Lumpy udder with one lump, tissue pliable and regular
6) Lumpy udder with two or more lumps, tissue pliable and regular

7) Lumpy udder with one lump, tissue non-pliable and irregularly shaped
8) Lumpy udder with two or more lumps, tissue non-pliable and irregularly shaped
9) Absence of significant mammary tissue on one side
10) Fibrous, leathery tissue, smaller than normal on one side
11) Hard mass in teat canal and cistern
12) Large hard mass
13) Abscess

Udder scores of 1 were omitted from analysis for lack of applicable information. Scores of 2 were categorized as favorable for this analysis. Scores greater than 2 were categorized as unfavorable since culling was usually performed to remove ewes with scores greater than 2.

Wool fleece was sheared annually, and fleece weight was greasy (no washing performed) weight in pounds.

Number of lambs born, number born alive, and number born dead were cumulative lifetime counts.

Ewe lifetime weight of lambs born were unadjusted in kilograms and ewe lifetime weight of lambs weaned were adjusted to an average age of 120 days in kilograms.

Table S1: *ZNF389* deletion variant genotype counts among lifetime SRLV-negative ewes.

Insertion Homozygote	Heterozygote	Deletion Homozygote	Total	Mean Age
73	183	55	311	3.89 years

Table S2: Association results between *ZNF389* deletion variant and production phenotypes in lifetime SRLV-negative ewes.

			**Adjusted means, by genotype**		
**Phenotype**	**Bonferroni P-value**	**Nominal P-value**	**Insertion Homozygote**	**Heterozygote**	**Deletion Homozygote**
**Birth weight (kg)**	1.00	0.41	NS	NS	NS
**Weaning weight (kg)**	1.00	0.085	NS	NS	NS
**Mature weight, 3 yr**	1.00	0.68	NS	NS	NS
**Mature weight, 4 yr**	1.00	0.84	NS	NS	NS
**Fleece weight (kg)**	1.00	0.36	NS	NS	NS
**Number lambs born**	1.00	0.51	NS	NS	NS
**Number lambs born alive**	1.00	0.52	NS	NS	NS
**Number lambs born dead**	1.00	0.99	NS	NS	NS
**Weight of lambs born (kg)**	1.00	0.097	NS	NS	NS
**Weight of lambs weaned (kg)**	1.00	0.13	NS	NS	NS
**Milk score**	1.00	0.53	NS	NS	NS
**Udder score, 3 yr**	1.00	0.90	NS	NS	NS
**Udder score, 4 yr**	1.00	0.69	NS	NS	NS

NS: Not nominally significant, P>0.05

Table S3: *ZNF389* deletion variant genotype counts among partial-lifetime group SRLV-negative ewes.

**Insertion Homozygote**	**Heterozygote**	**Deletion Homozygote**	**Total**	**Mean Age**
81	261	111	453	3.49 years

Table S4: Association results between *ZNF389* deletion variant and production phenotypes among partial-lifetime group SRLV-negative ewes.

			Adjusted means, by genotype		
**Phenotype**	**Bonferroni P-value**	**Nominal P-value**	**Insertion Homozygote**	**Heterozygote**	**Deletion Homozygote**
**Birth weight (kg)**	0.033	0.0025	4.67	4.88	5.08
**Weaning weight (kg)**	1.00	0.33	NS	NS	NS
**Mature weight, 3 yr**	1.00	0.19	NS	NS	NS
**Mature weight, 4 yr**	1.00	0.44	NS	NS	NS
**Fleece weight (kg)**	0.088	0.0068	15.17	16.51	17.06
**Number lambs born**	1.00	0.91	NS	NS	NS
**Number lambs born alive**	1.00	1.00	NS	NS	NS
**Number lambs born dead**	1.00	0.70	NS	NS	NS
**Weight of lambs born (kg)**	1.00	0.55	NS	NS	NS
**Weight of lambs weaned (kg)**	1.00	0.90	NS	NS	NS
**Milk score**	1.00	0.85	NS	NS	NS
**Udder score, 3 yr**	1.00	0.97	NS	NS	NS
**Udder score, 4 yr**	1.00	0.95	NS	NS	NS

NS: Not nominally significant, P>0.05
